# Direct flow automated serum-iron determination

**DOI:** 10.1155/S1463924682000066

**Published:** 1982

**Authors:** Ferruccio Ceriotti, Piero Bonvicini

**Affiliations:** Laboratorio Centrale-Ospedale Civile di Padova Padova 35100 Italy


Journal of Automatic Chemistry, Volume 4, Number 1 (January–March 1982), pages 17–20

### Direct flow automated serum-iron determination

**Ferruccio Ceriotti and Piero Bonvicini**

*Laboratorio Centrale-Ospedale Civile di Padova, 35100 Padova, Italy*

### Introduction

The major difficulties of the direct methods for serum-iron determination lie in the possible formation of unpredictable, and often progressive, turbidity and in the sometimes incomplete release of iron from transferrin. Another important drawback, which the direct methods share with methods involving deproteinization (except atomic absorption), is a lack of specificity and accuracy due to variable copper interference with the chromogenic complexing agents, especially the highly sensitive ferrozine.

All these drawbacks have apparently been overcome in a recent method [1], which simply chelates iron with ferrozine at a low pH, in the presence of dilute hydrochloric acid, ascorbic acid and thiosemicarbazide. This method completely blocks copper as an uncoloured complex, without affecting the reaction of iron with ferrozine.

The new method can be used with continuous flow automation without dialysis. Some difficulties have been found, mainly due to the adsorption of iron from aqueous solutions on tygon tubes. In this paper experiments related to this phenomenon are described and the method that was finally adopted at the Ospedale Civile di Padova is discussed.

### Experimental

### *Materials*

The following materials were used: 3-(2-pyridyl)-5,6-diphenyl-1,2,4-triazine, sulphonic acid disodium salt (ferrozine) (supplied by Aldrich); iron-free ascorbic acid (supplied by Merck); thiosemicarbazide (supplied by Carlo Erba); glycine (Merck); calibrated ferric chloride solution (1 g of iron/l) in 1 mol/l of hydrochloric acid: 'Titrisol iron' (Merck).

### *Solutions*

(1) The reagent for the blank was prepared by dissolving 2 g of ascorbic acid and 100 mg of thiosemicarbazide in 200 ml of hydrochloric acid (0·05 mol/l), finally 1 ml of Brij 35 (30% solution) was added.
(2) The chromogenic reagent consisted of 50 mg of ferrozine dissolved in 100 ml of reagent 1. This reagent is stable for three hours at room temperature and for about 16 h at 4°C.
(3) Wash solutions were sodium citrate (0·1 mol/l), and sodium chloride (0·15 mol/l).
(4) For reasons discussed later in this paper, serum is preferred as the standard. The iron content of a pool of sera is determined by the manual method [1] and eventually taken to the desired value (i.e. 27 μmol/l) by addition of a calibrated solution of ferric iron, determined again, distributed in ampules and stored frozen. Also some commercial sera (for example, Precinorm, Precipath, Sclavo, Ortho) have been tested and found to be suitable as standards.

### *Procedure*

The determination is performed on an Autoanalyser II (manufactured by Technicon) using the very simple manifold illustrated in figure 1.

The chromogenic reaction and the blank are processed simultaneously, the two fluxes are phased and the blank value is automatically substracted by a double-beam colorimeter, which has an interference filter set at 560 nm. Phasing is easy to obtain because of the short dwell time. A speed of 60 samples/h has been found convenient with a sample-to-wash ratio of 2 : 1; the total

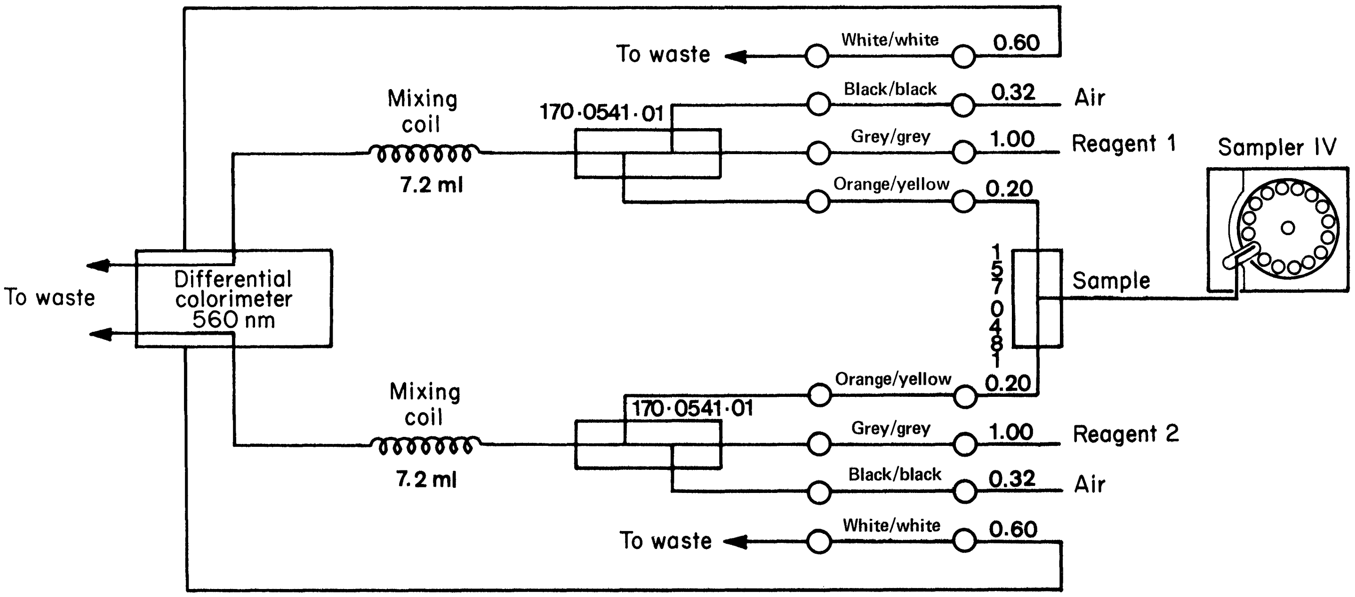

*Figure 1. Flow diagram for direct determination of serum iron.*

0142-0453/82/0401 0017 $05·00 © 1982 Taylor & Francis Ltd sample volume utilized is 300 μl and the final sample dilution is 1 to 5. Before starting a series of determinations, the apparatus is primed by sampling the citrate solution twice and the saline solution once.

### *Results*

The possibility of turbidity formation, which is easily detectable through the characteristic noise it gives on the recorder, has been checked using plasma from 10 myeloma and 20 cirrhotic patients with $\gamma$ globulin concentrations ranging from 32 to 57 g/l. No turbidity was observed, despite these challenging plasmas.

Intra-assay repeatability was tested on sera at three different concentrations (table 1). The highest coefficient of variance was 1·36. The inter-assay reproducibility was also tested at three iron levels. The results are shown in table 2. The highest coefficient of variance was 3·12 for an iron concentration of 8·95 μmol/l.

*Table 1. Test of intra-assay repeatability on sera at three serum-iron levels.*

| *N* | $\bar{x}$ μmol/l | Standard deviation | Coefficient of variance |
|---|---|---|---|
| 30 | 11·1 | ∓0·125 | 1·13 |
| 11 | 15·75 | ∓0·215 | 1·36 |
| 31 | 43·3 | ∓0·286 | 0·66 |

*Table 2. Test of inter-assay reproducibility at three serum iron levels.*

| *N* | $\bar{x}$ μmol/l | Standard deviation | Coefficient of variance |
|---|---|---|---|
| 20 | 8·95 | ∓0·28 | 3·12 |
| 20 | 16·3 | ∓0·37 | 2·26 |
| 20 | 43·22 | ∓0·45 | 1·04 |

Carry-over between sera was tested at concentrations of 43·86, 14·32 and 7·16 μmol/l. The testing sequence was three high-concentration samples followed by three low-concentration samples.

Each experiment was repeated three times and calculations were made according to the following formulae:

$$\frac{\text{low}_1 - \text{low}_3}{\text{high}_3 - \text{low}_3} \times 100$$

for the high to low sequence, and

$$\frac{\text{high}_3 - \text{high}_1}{\text{high}_3 - \text{low}_3} \times 100$$

for the low to high concentration. A mean contamination of 2·1% from low to high and of 2·2% from high to low was obtained.

At a lower sampling speed, namely 50 samples/h, a lower contamination of about 1% was observed.

The peaks reach 96% and 97·5% of the steady-state height for a sampling speed of 60 and 50 samples/h respectively, at a serum-iron concentration of 35·8 μmol/l.

The linearity of the reaction has been verified up to 90 μmol/l. For routine determinations, sensitivity was set at full recorder scale deflection for a concentration of 50 μmol/l. This covers almost all the values found in practice and allows good resolution at low concentrations.

The elimination of copper interference by addition of thiosemicarbazide, although already proved in the manual method, was tested again in the continuous-flow procedure. Copper was added to serum up to a concentration of 785 μmol/l without any alteration in the iron values.

Recovery experiments were performed by adding four different concentrations of a calibrated iron solution to serum. As table 3 shows, a mean recovery of 100·05% was observed.

*Table 3. Recovery experiments.*

| μmol/l | | | | |
|---|---|---|---|---|
| Additions | Theor. | Found | Recovered | Recovery% |
| | | 9·58 | | |
| 8·95 | 18·53 | 18·20 | 8·62 | 96·3 |
| 17·90 | 27·48 | 27·00 | 17·42 | 97·3 |
| 26·85 | 36·43 | 37·40 | 27·82 | 103·6 |
| 35·80 | 45·38 | 46·5 | 36·92 | 103·0 |
| | | | Mean recovery | 100·05 |

### *Preparation of standards*

Aqueous standards were prepared initially in the same way as the standards used in the manual method: calibrated Titrisol iron solution was diluted in 0·12 mol/l of glycine buffer at pH 2·2. However, unlike the results obtained with sera, reproducibility was poor. This phenomenon was systematically investigated and it was found that when making a series of determinations of the same aqueous standard, there was a progressive increase of the height of the peaks up to a constant value. It was also noted that the first serum following an aqueous standard presented a very sharp and high peak that was absent in subsequent sera (see figure 2 [*a*]). A similar, but lower, peak was discovered in the first serum sample after a sequence of washings of the system with

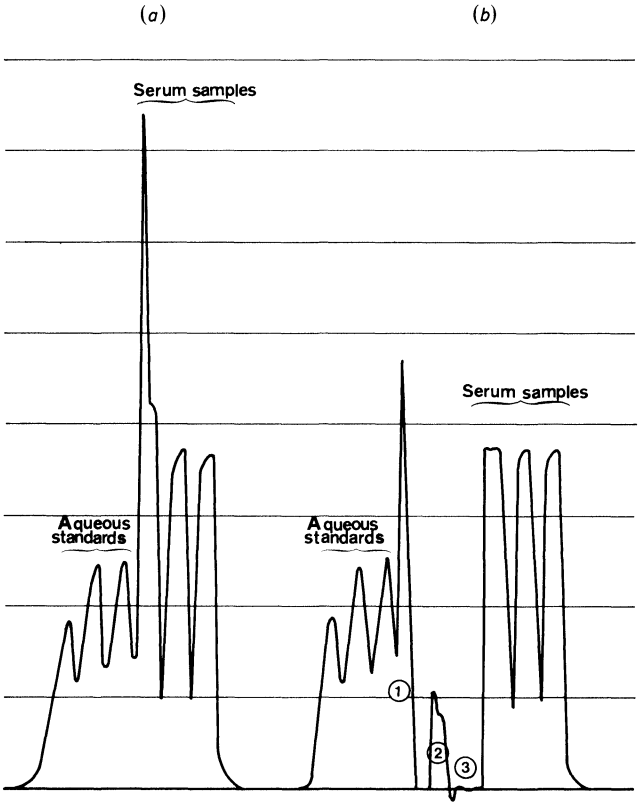

*Figure 2(a). Effect of iron standards (17·9 μmol/l) in 0·12 mol/l glycine buffer (at pH 2·2) on the first subsequent serum sample. (b) Elimination of the peak by two samplings of sodium citrate (0·13 mol/l) (1 and 2) and one sampling of water (3).*

water. This phenomenon varied in intensity with various sets of tubes, but was always present. The only explanation for it was adsorption of iron from aqueous solutions on the internal walls of the tygon tubes followed by its capture by unsaturated transferrin in the subsequent serum. Two trials were carried out to test this hypothesis. The first was to eliminate the peak in serum samples subsequent to aqueous iron solutions by washings with displacing agents; and the second to prepare aqueous iron solutions free from adsorption phenomena. Displacing agents used in the first trial were complexing substances and concentrated salt solutions.

The complexing substances in the first trial were sodium citrate (0·13 mol/l and 5 mmol/l); ferrozine (0·5 g/l in 20 g/l ascorbic-acid solution); and EDTA tripotassium salt (0·4 mol/l and 4 mmol/l). The concentrated salt solutions used were acetate buffer at pH 5·2, 2 mol/l, and sodium chloride (2 mol/l). These solutions were sampled twice after a series of three or more aqueous standards (17·9 μmol/l of iron at pH 2·2, 0·12 mol/l using glycine buffer).

The peak in the subsequent serum was eliminated by the more concentrated citrate (figure 2 [*b*]) and EDTA, and by the acetate and sodium-chloride solutions. The less concentrated EDTA and citrate cleared the tubes only partially, as did the ferrozine (figure 3 [*b*]). When the more concentrated EDTA was used, the serum peaks were lower than expected (figure 3 [*a*]). After these experiments, sodium citrate and EDTA were added to the iron solution in glycine buffer up to concentrations of 50 μmol/l and 40 μmol/l, respectively. The chromogenic reaction with ferrozine was partially inhibited by citrate and completely by EDTA; in both cases some iron remained adsorbed on the tubes, as proved by the height of the peak for the first subsequent serum sample (see figures 4 [*a*] and 4 [*b*]). The best results were obtained by diluting the standard iron titrisol solution in 2 mol/l of acetate buffer at pH 5·2 and in 2 mol/l of sodium chloride at pH 2·2, with 0·12 mol/l glycine buffer. The reaction developed completely; but an increase, albeit very small, in the first subsequent serum peak revealed that traces of iron had remained adsorbed on the tubes (figure 5).

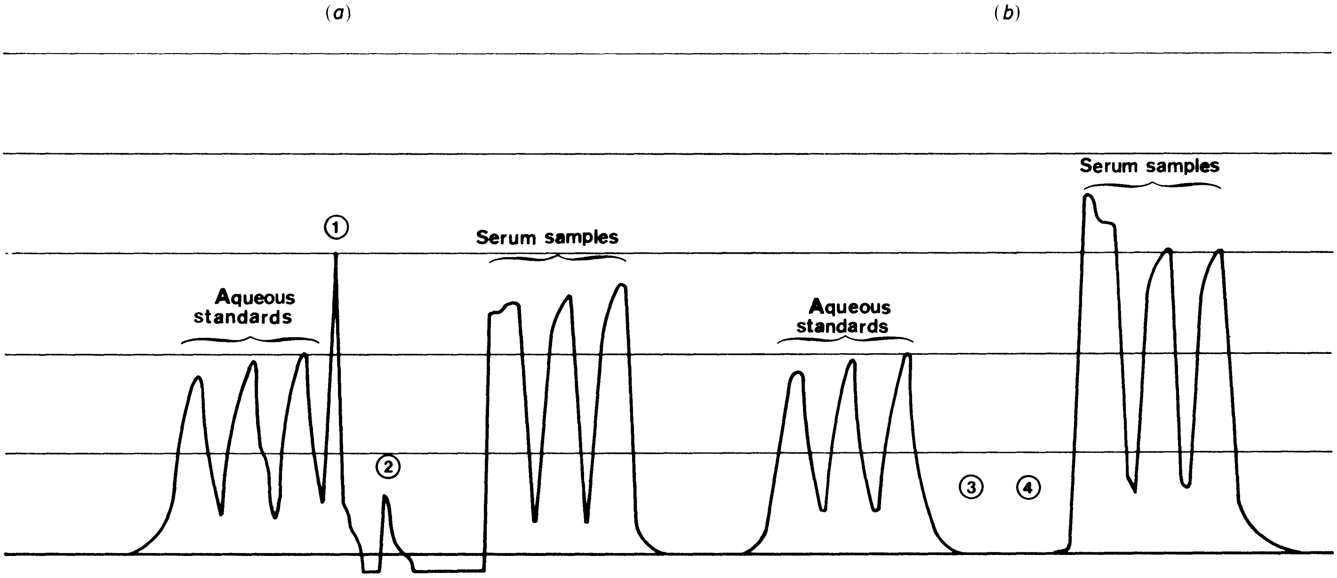

*Figure 3. Desorption of iron adsorbed on tubes from aqueous standards by sampling with EDTA 4 mmol/l ([a]: 1 and 2) and 0·4 mol/l ([b]: 3 and 4). The same serum has been used in both cases. The peak in the first serum is eliminated and the serum peaks decreased in figure 3 (a). In (b), the first serum sample shows the increase due to the capture by transferrin of the iron not desorbed from tubes by the dilute EDTA.*

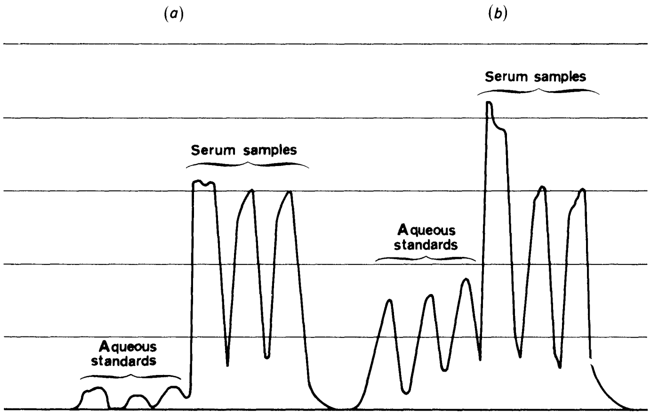

*Figure 4 (a). Addition of EDTA (40 μmol/l) to aqueous iron standards (17·9 μmol/l). The binding of iron with EDTA inhibits its reaction with ferrozine and prevents its adsorption on tubes as proved by the normal appearance of the subsequent sera. (b) Addition of citrate (50 μmol/l). The reaction with ferrozine is only partially inhibited and the iron is adsorbed on tubes as shown by the peak of the first subsequent serum.*

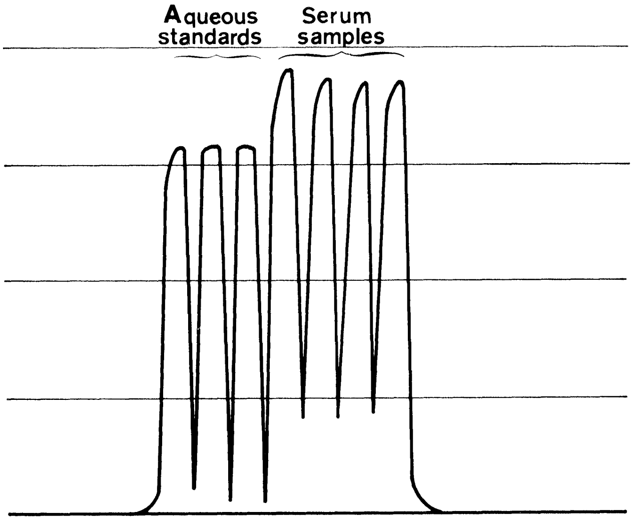

*Figure 5. Iron standard (17·9 μmol/l) in 2 mol/l of sodium acetate buffer at pH 5·2. A very small adsorption of iron on tubes is detectable by slightly increased height of the subsequent serum peak.*

The effect of increasing concentrations of bovine albumin on the adsorption of iron from aqueous solutions was then tested. The affinity of iron for albumin appeared to be stronger than that of the tygon tubing, and at an albumin concentration of 8·0 g/l no more iron was adsorbed (figure 6).

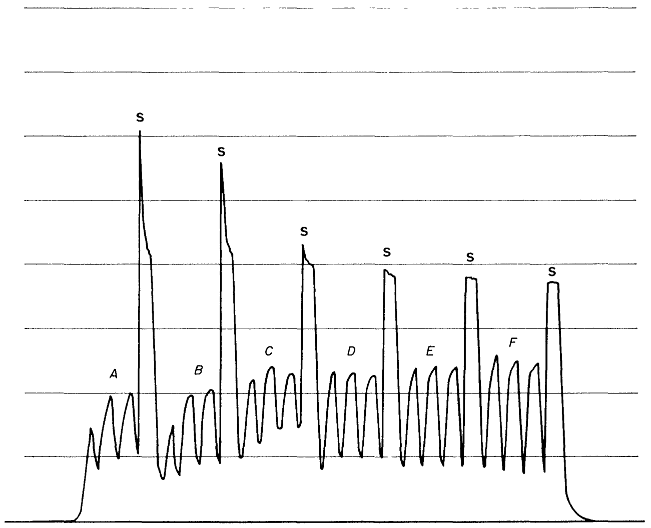

*Figure 6. Effect of increasing concentrations (a: 1·0, b: 2·0, c: 8·0, e: 12·0 and f: 20·0 g/l) of bovine serum albumin in standard iron solutions (17·9 μmol/l in 0·12 mol/l, and glycine buffer at pH 2·2). The adsorption of iron on tubes is progressively decreased and finally eliminated as shown by the decrease and disappearance of the peak of the subsequent serum samples (S).*

### Discussion

The principles underlying the manual methods for determination of serum iron, namely the performance of the iron-ferrozine reaction at a low pH, in dilute hydrochloric acid and in the presence of thiosemicarbazide and ascorbic acid, have been demonstrated to apply to a flow automated system without dialysis. The results using continuous flow automation show an improvement over the direct method in the absence of turbidity even in plasma samples with high levels of paraproteins, copper interference has been eliminated, and there is rapid release of iron from transferrin and thus complete formation of the coloured complex.

The reaction was performed at a higher serum dilution than in the manual procedure. The sensitivity of the automated system is high enough to allow the use of relatively small amounts of serum (150 μl); dilution is then necessary to attain a reasonably low viscosity and to reduce the carry-over to a minimum when the sampling speed is high. The concentration of hydrochloric acid is lower in the automated procedure than it is in the manual one, to allow for the reduced protein concentration. The ratio of hydrochloric acid to protein has to be maintained, however, to give a final pH within the optimal range for colour development.

An unexpected finding was the adsorbance of iron from aqueous solutions onto the walls of the tygon tubes. The affinity of iron for the tube is lower than for transferrin, therefore the first part of a following serum acts as a scavenger for protein adsorbed from the previous sample, as shown by a very sharp and thin peak. Albumin has superior affinity for iron than does tygon tubing, when added in sufficient concentration, it prevents the adsorption of iron on tubes. A concentrated acetate buffer can almost completely prevent this phenomenon, probably by an ion-exchange process.

Albumin solutions can therefore be used to prepare standard solutions, but they do not seem to offer any advantages over sera. In fact, for maximum efficiency, albumin must be used in rather high concentrations and has the disadvantage of containing varying amounts of iron. So albumin solutions cannot be considered as primary standards and they must always be checked to determine their true iron content in the same way that sera have to be checked. Sera solutions have the advantage of possessing matrices identical to the samples, they are always at hand and are easy to prepare and are inexpensive. Commercial sera have also proved to be usable; first their iron content has to be determined and their possible turbidity checked by the manual method.

It was found convenient, when starting a series of tests, to prime the apparatus by two samplings of citrate solution followed by a sampling of saline. This is necessary to clear the tubes from traces of iron left by washing the apparatus with water. A simpler alternative is to start with more than one serum standard using the first one as a scavenger.

The most important feature of the present method is the elimination of dialysis. Dialysis has always constituted a critical step in automated flow methods for the determination of iron [2, 3, 4]. The dialysis rate of iron is low and this requires a large dialysing surface, a large sample to account for the low sensitivity, and the addition of pepsin, to keep the dialysis membranes clean. Furthermore, according to Thiers [3], since the solution is viscous, the fluid has to be pumped out of the dialyser in order to keep the hydrostatic pressure within reasonable limits and to produce uniform flow. Dwell time in the dialyser is hard to predict under these conditions.

All these drawbacks are avoided in the present method and a small sample can be used. Other direct methods are difficult to apply to continuous flow automation because problems of flow may arise from the large concentrations of surfactants to be used.

Application of the present method to discrete and parallel automated systems is now under study.

### References

1. Ceriotti, F. and Ceriotti, G., *Clinical Chemistry*, **26** (1980), 327.

2. Zak, B. and Epstein, E., *Clinical Chemistry*, **11** (1965), 64.

3. Thiers, R. E., in *Clinical Chemistry Principles and Techniques*, Ed. Henry, R. J., Cannon, D. C., and Winkelman, J. W. (Harper and Row, London, 1974), p. 225.

4. Weissman, N. and Pileggi, V. J., in *Clinical Chemistry—Principles and Techniques*, op. cit., p. 686.

